# Visual Attention Patterns Differ in Dog vs. Cat Interactions With Children With Typical Development or Autism Spectrum Disorders

**DOI:** 10.3389/fpsyg.2020.02047

**Published:** 2020-09-04

**Authors:** Marine Grandgeorge, Yentl Gautier, Yannig Bourreau, Heloise Mossu, Martine Hausberger

**Affiliations:** Univ Rennes, Normandie Univ, CNRS, EthoS (Éthologie animale et humaine) – UMR 6552, Rennes, France

**Keywords:** human–pet interactions, social visual attention, autism, dogs, cats

## Abstract

Visual social attention is an important part of the social life of many species, including humans, but its patterning may vary between species. Studies on human–pet relationships have revealed that visual attention is also part of such interspecific interactions and that pets are sensitive to the human visual attentional state. It has been argued that domestication and/or repeated experiences with humans have shaped and refined these decoding abilities. Little is known on how the species’ evolutionary history may play a role in determining visual attention patterns during interactions, nor how the human’s own social skills may influence the animal’s attention patterns in human–animal interactions. In the present study, we investigated the visual attention patterns directed to the partner in dog–child and cat–child interactions in their home environment. We also compared these patterns between a group of children with autism spectrum disorders (ASD) and children with typical development. We found that the attention patterns differed according to species, with dogs displaying more gazes and cats more glances toward their human interlocutor, while children showed gazes toward both species. Only slight differences were observed according to the developmental status of children: ASD children displayed much more visual attention with their pet cat than with their pet dog and the same amount of visual attention toward their pet, whatever the species, as typically developing (TD) children. Because humans rely a lot upon visual communication in their own social encounters, where direct gazes play a major role from early on, they may be especially sensitive to the gazing behavior of their dogs. People with ASD, with a less typical pattern of interaction, may be more comfortable with the less “invasive” short glances of cats. These results suggest not only that interspecific communication has to be associated with processing and storing the other species’ ways of communicating in order to be successful but also that visual attention patterns during interactions, even when interspecific, are, for a large part, the result of the species’ own evolutionary history.

## Introduction

Social attention is one of the most important aspects of social life (Scheid et al., 2007), and according to Goffman (1961), attention is what makes the difference between a proper social interaction and a mere co-presence. Visual attention is in this regard an important component of social interaction in a variety of species (Snowdon and Hausberger, 1997; Lemasson and Hausberger, 2004). The pattern of visual attention may vary according to the species’ social characteristics, from a “dominant-centered” visual focus to a “monitor-adjust” system of divided attention toward all group members in tolerant species (e.g., York and Rowell, 1988; Blois-Heulin and Girona, 1999; Lemasson et al., 2006). In corvids, jackdaws show more attention toward non-affiliates, whereas ravens spend more time gazing at friends (Scheid et al., 2007). There has been growing evidence that, in many species, far from being a mere threat signal as long thought, social gazing, and gaze following may reflect friendship (Hattori et al., 2007; Micheletta and Waller, 2012). In primates, affiliative and status relationships do affect how much individuals attend visually to others (Chance and Joly, 1970). Attention patterns also vary between species and according to context, favoring either short glances or durable gazes toward group members (Day et al., 2003). In humans, longer gazes (more than 1 s) correspond to the shift from a common focus on a topic of interest to a focused attention to the interlocutor, especially in the case of affiliative attraction (Kendon, 1967).

Visual attention is also an important part of interspecific communication, as observed in human–animal interactions. Domestic, but also captive wild animals, have been shown to present a sensitivity to human attentional states, especially through gaze direction (e.g., dogs: Call et al., 2003; Schwab and Huber, 2006; horses: Sankey et al., 2011; capuchin monkeys: Hattori et al., 2007; red-capped mangabeys: Maille et al., 2012). These abilities may reflect, in the first case, effects of domestication, i.e., selection of animals more skilled in decoding human cues, but also, in both domestic and wild captive animals, shared experiences during repeated interactions (e.g., Leroux et al., 2018). Humans also are very sensitive to their pet’s visual attention, as shown recently: dog owners exhibit an increase of oxytocin as a result of their dogs showing long gazes toward them (Nagasawa et al., 2015). Although the sensitivity to human attentional state is widespread among domestic animals, the extent of this ability is different according to species and may well depend upon the evolutionary processes underlying the species’ own social dispositions (Chance and Joly, 1970; Mason, 1978). Dogs and cats, for example, although both companion animals, differ in their level of distractibility, which could be explained by a better inhibitory control of cats which have a “sit and wait” predatory strategy (Kraus et al., 2014). Social canids may, on the other hand, rely upon visual contact between group members for hunting (e.g., Bekoff et al., 1984). In dogs, sustained gazes may reflect attempts of dominance (“staring”), but also affiliative behaviors (e.g., Bradshaw and Nott, 1995).

To our knowledge, there is no scientific information about how these two companion species differ in terms of visual attention in spontaneous interactions with humans. On the other hand, the human responses themselves may both depend upon the pet’s behavior and their own human’s attentional skills. Human infants, from the first days of life, follow other people’s gaze and seek eye contact and mutual gazing, which are crucial for their development and long-term parent–child bonding (Scaife and Bruner, 1975; Farroni et al., 2002; Guellai et al., 2014).

However, social visual attention is impaired in some types of atypical development, e.g., autism spectrum disorders (ASD) (Goldstein et al., 2001; APA, 2013). People with ASD have difficulties in the perception of direct and indirect human gazes (Forgeot d’Arc et al., 2017) and a limited use of mutual gaze or joint attention during interactions with peers (Emery, 2000). They also display an atypical visual exploration of human face pictures, focusing mainly on the mouth part (Guillon et al., 2014). They show an increased arousal when submitted to a human direct gaze (O’Haire et al., 2015). Interestingly, a recent eye tracking study shows that ASD children look at eyes when animal faces are presented (Grandgeorge et al., 2016), as also suggested by numerous testimonies (Grandin and Johnson, 2005).

In the present study, we hypothesized that the visual attention patterns would differ during dog–child and cat–child interactions due to species differences in attentional and bonding characteristics. Dogs, as a social cooperative canid, are expected to exhibit more durable gazes and cats, as a solitary opportunistic gregarious species, more short glances. Moreover, we also investigated the impact of the human interlocutor *per se* on the dyad’s pattern of visual attention by comparing a group involving children with typical development to another group involving ASD children, i.e., with altered visual social skills. Observations were performed in the home environment so as to have “ecological” data from already bonded interspecific partners. Questionnaires allowed us to additionally have the parents’ perception of the quality of their child’s interactions and bonding with their pet animal.

## Materials and Methods

### Ethical Concern

Data were collected between 2009 and 2012, in accordance with the (at that time) current French legislation. As this research was observational, it required no ethics committee at this time. All the dogs and cats involved in the study were family pets, hence under their owners’ responsibility for care and use. The researchers had no involvement in any decision in this regard, and the study was conducted in accordance with the French regulations governing the use of animals for research. Regarding humans, all parents provided free, informed, and written consent for the participation of their child in the study, all in accordance with the Declaration of Helsinki (6th revision), and French regulations at that time. The parents gave their written consent to allow us to film their child.

### Participants

#### General Information

Forty-two children were recruited: they were all aged 6–12 years, had no prior parent-reported history of animal abuse, and had no physical disability that could limit their interactions with their dog or cat. Nineteen children with typical development were included after recruiting by adverts. Twenty-three children with ASD came from the “Centre de Ressources sur l’Autisme de Bretagne,” CHRU Brest, Bohars, France. Behavioral assessments were performed using the Autism Diagnostic Interview – Revised (ADI-R) (Lord et al., 1994). The ADI-R, an extensive, semi-structured parental interview, was conducted by independent psychiatrists. The ADI-R scale assessed the three major domains of autistic impairments: reciprocal social interactions, verbal and non-verbal communication, and stereotypic behaviors and restricted interests. Based on direct clinical observations of each child by independent child psychiatrists, a diagnosis of ASD was made according to the DSM-IV (APA, 2000) and ICD-10 (World Health Organization, 1994) criteria and was confirmed by the ADI-R ratings. Table 1 presents the sample populations.

**TABLE 1:** Characteristics of the pet population (young = under 1 year of age).

	Pet dog	Pet cat
*N*	26	16
Gender (M/F)	16/10	9/7
Age (young/adult)	0/26	3/13
Purebred/mixed breed/mongrels	22/0/4	3/0/13
Details about breeds	Large-sized mongrels, *n* = 1	Chartreux, *n* = 1
	Middle-sized mongrels, *n* = 3	Siamese, *n* = 2
	Border Collie, *n* = 1	Mongrels, *n* = 13
	Bernese mountain dog, *n* = 1	
	Boxer, *n* = 2	
	Cavalier King Charles spaniel, *n* = 3	
	Cocker spaniel, *n* = 1	
	Golden retriever, *n* = 3	
	Groenendael, *n* = 2	
	Jack Russel Terrier, *n* = 2	
	Labrador retriever, *n* = 4	
	Lhasa Apso, *n* = 1	
	Newfoundland dog, *n* = 1	
	Yorkshire terrier, *n* = 1	

Because the quality of the relationship may influence the pattern of visual attention, we used a parent-based short questionnaire to have an evaluation of it (see also Grandgeorge et al., 2012). This was represented by two items: information about the frequency of visual interaction between their child and their pet (according to a three-point Likert-scale: never, rarely, and often) and whether they considered the child–pet relationship as a “privileged” relationship, such as “favorite pet of the child, spending time and playing together and reciprocal behaviors”) (defined by Grandgeorge et al., 2014).

#### Population 1: Pet Dogs and Associated Children

The population of pet dogs included 16 males (eight with children with ASD and eight with children with typical development) and 10 females (six with children with ASD and four with children with typical development), four mongrels and 22 purebreds, all adults (more than 18 months old) (Table 1).

The 26 children involved (Table 2) consisted of 14 children with ASD, all males (mean age = 10.1 ± 2.1 months), matched on chronological age with 12 children with typical development (eight boys and four girls, mean age = 9.4 ± 2.4 years) [Mann–Whitney test: *U*_(14, 12)_ = 75.5, *p* = 0.680].

**TABLE 2:** Characteristics of the sample population of children.

	Living with pet dogs	Living with pet cats
**Characteristics of the ASD children**		
*N*	14	8
Mean age ± SD (in years)	10.1 ± 2.1	7.5 ± 2.2
Gender (M/F)	14/0	8/0
Privileged relationships (yes/no)	8/6	8/0
Frequency of visual interaction (according to parents)	7 often 5 rarely 2 “don’t know”	4 often 3 rarely 1 “don’t know”
**Characteristics of the children with typical development**		
*N*	12	8
Mean age ± SD (in years)	9.4 ± 2.4	9.0 ± 1.9
Gender (M/F)	8/4	3/5
Privileged relationships (yes/no)	9/3	6/2
Frequency of visual interaction (according to parents)	9 often 2 rarely 1 never	5 often 2 rarely 1 “don’t know”

#### Population 2: Pet Cats and the Associated Children

Sixteen pet cats were involved, corresponding to nine males (three with children with ASD and six with children with typical development) and seven females (five with children with ASD and two with children with typical development). Thirteen were mongrels and three others were purebred. Three were less than 1 year old (i.e., young, all with children with ASD) and 13 were adults (Table 1).

The 16 children (Table 2) corresponded to eight children with ASD, all males (mean age = 7.5 ± 2.2 years), matched on age with eight children with typical development (three boys and five girls, mean age = 9.0 ± 1.9 years) [Mann–Whitney test: *U*_(8, 8)_ = 13, *p* = 0.160].

### Experimental Design

One-hour observation sessions were performed at the child’s home. They were performed at fixed times (4–6 p.m.), when the children were back from school or institution. Before starting, the observer (MG) asked the child and the other people present (e.g., father, mother, and siblings) to behave as usual and made clear that no behavior was considered either good or bad. She carried a camera and filmed the child’s behavior continuously (including interactions with the pet or with family members). She remained silent and did not take part in the interactions (MacGrew, 1972; Millot et al., 1988).

### Data Collection

Behavioral data were sampled from the video recordings using continuous focal sampling. Behavioral data were only analyzed when both child and pet were visible on the video recording (Altmann, 1974). Different parameters of pet and child visual attention were measured and the initiator of the visual interaction was identified. Thus, occurrences and, when appropriate, durations (in seconds) were calculated for the following behavioral items:

• Glances: focusing eyes on the other partner at ± 5° for less than 1 s (Blois-Heulin and Girona, 1999).
• Gazes: focusing eyes on the other partner at ± 5° for at least 1 s.
• Mutual gazes: child’s and pet’s attention was directed to one another (Emery, 2000).

According to Emery (2000), several cues could be used to determine the direction of visual attention: when the eyes were little or not visible, the orientation of the head and/or body was used. If the eye direction was not clearly identified, it was recorded as non-visible.

All data analyses were performed by three observers (YG, YB, and HM), blind to the child diagnosis. Inter-observer reliability was ensured by training with one senior author (MG) until full agreement was reached.

### Statistical Analyses

As data were not normally distributed, we used non-parametric statistical tests (Siegel and Castellan, 1988). As the duration of the children–pet visibility varied between video recordings, we homogenized all data by calculating the number of occurrences and the durations per minute. Mann–Whitney *U* tests were used to compare two independent samples (e.g., gaze duration toward the pet between the two groups of children). Wilcoxon signed-rank tests were used to compare dependent samples (e.g., children’s gaze duration toward the pets compared to pet’s gaze duration toward the same children). Spearman’s tests were used to evaluate the correlations (e.g., between children’s gaze occurrences and dog’s gaze occurrences). These analyses were run with Statistica software and R software with an accepted *p* level at 0.05.

## Results

### Visual Attention Between Dogs and Children

#### Pet Dogs

During the observation sessions, the child–dog dyads were visible around 50% of the video recording, whatever the child’s status (ASD: 1,562.46 ± 937.23 s, TD: 2,084.67 ± 821.98 s, respectively, *U* = 58, *p* = 0.189).

**FIGURE 1:**
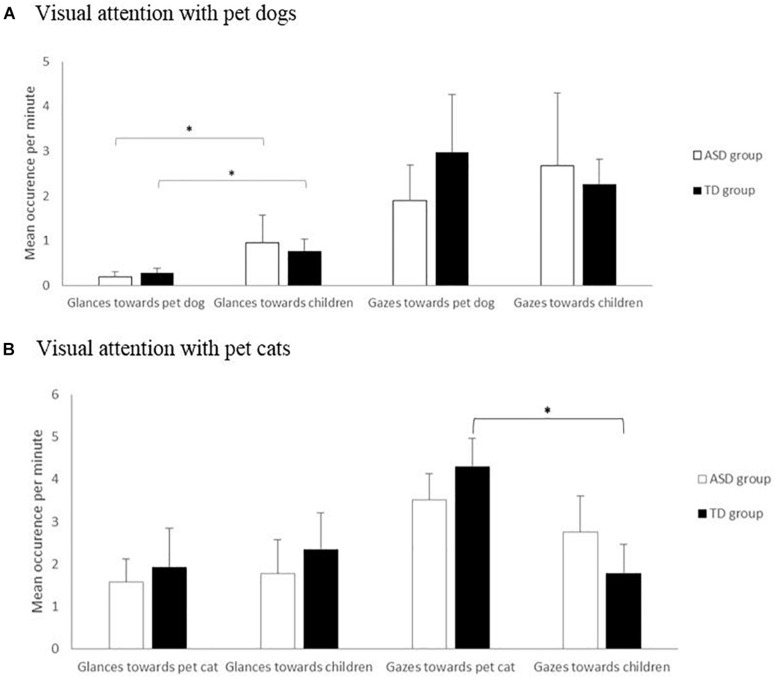
Visual attention in the autism spectrum disorders (ASD) and typically developing (TD) groups in occurrence per minute (glances and gazes) in pet dog–child **(A)** and pet cat–child **(B)** dyads. Mann–Whitney and Wilcoxon tests. ^∗^*p* < 0.05.

Overall, there was no difference in the structure of visual attention between the TD and ASD groups for both children and pets (all Mann–Whitney tests: *p* > 0.05; Figure 1A): all dogs and children performed more gazes (mean occurrence: dogs, 2.48 ± 2.47 per minute; children, 2.38 ± 2.14 per minute) than glances (mean occurrence: dogs, 0.80 ± 1.18 per minute; children, 0.24 ± 0.19 per minute; *Z* = 4.371 and 4.284, respectively, *p* < 0.001).

In both groups, pet dogs more frequently initiated glances (but not gazes) toward the children than did the children toward the dogs (ASD group: 0.96 ± 1.55 vs. 0.2 ± 0.19 per minute, *W* = 2.417, *p* = 0.016; TD group: 0.61 ± 0.51 vs. 0.29 ± 0.19 per minute, *W* = 2.045, *p* = 0.041) (Figure 1A). Moreover, the visual attention displayed by the dogs and children were not correlated (whatever the children group, visual attention type, and measures; all Spearman’s correlation, *p* > 0.05). Neither the frequency nor the duration of dog–child mutual gazes differed between groups (occurrence: ASD, 2.28 ± 3.18 s; TD, 2.34 ± 2.7 s, *U* = 66, *p* = 0.374; duration: ASD, 4.77 ± 3.44 s; TD, 5.55 ± 3.46 s, *U* = 71, *p* = 0.520, respectively).

#### Pet Cats

The cat–child dyads were visible only around 10% of the video recording for both the ASD and TD children groups (646.51 ± 335.90 and 605.53 ± 424.63 s, respectively, *U* = 25, *p* = 0.495).

Overall, the two groups did not differ in the structure of their visual attention: cats showed an equal proportion of glances and gazes in both cases (2.3 ± 1.6 and 2.1 ± 0.8 per minute, *Z* = 0.451, *p* = 0.649, respectively), whereas children – whatever their diagnostic group – displayed more gazes (mean occurrence: all children, 3.9 ± 1.3; ASD children, 3.5 ± 1.2; TD children, 4.3 ± 1.3 per minute) than glances (mean occurrence: all children, 1.8 ± 1.5; ASD children, 1.6 ± 1.1; TD children, 1.9 ± 1.8 per minute; *Z* = 3.244, *p* = 0.001) toward their pet. Mutual gazes were rare (mean occurrence, 0.7 ± 0.7 per minute; ASD children, 0.6 ± 0.6 per minute; TD children, 0.7 ± 0.8 per minute) and less frequent than unilateral gazes and glances both in children and pet cats (all Wilcoxon tests: *p* < 0.001).

Overall, there was no significant difference according to the child group, whether in children’s or pets’ attentional characteristics (glances, gazes, and mutual gazes; all tests: *p* > 0.05) (Figure 1B).

In both groups, the cats and children initiated glances (respectively, glances toward cats: for all children, 1.7 ± 1.5; for ASD children, 1.6 ± 1.1; for TD children, 1.9 ± 1.8 per minute; glances emitted by cats toward all children, 2.1 ± 1.6; toward ASD children, 1.8 ± 1.6; toward TD children, 2.3 ± 1.8 per minute) (Figure 1B) and mutual gazes (respectively, mutual gazes initiated by all children, 0.2 ± 0.3; by ASD children, 0.2 ± 0.3; by TD children, 0.2 ± 0.3; mutual gazes initiated by cats toward all children, 0.5 ± 0.5; toward ASD children, 0.4 ± 0.4; toward TD children, 0.5 ± 0.7 per minute (Figure 1B) equally often.

Finally, TD children initiated more frequent and longer gazes toward their pet cats than did their pet cats (4.3 ± 0.7 vs. 1.8 ± 0.7 per minute, *W* = 34, *p* = 0.023; 0.8 ± 0.5 vs. 0.3 ± 0.2 per minute, *W* = 35, *p* = 0.016) (Figure 1B), whereas no such difference was found in the ASD group (occurrence and duration: all Wilcoxon tests, *p* > 0.05). However, the visual attention displayed by the cats and children were not correlated (whatever the children group, visual attention type, and measures: all Spearman’s correlation, *p* > 0.05).

### Differences of Visual Attention Patterns Between Pet Dog–Child Dyads vs. Pet Cat–Child Dyads

**FIGURE 2:**
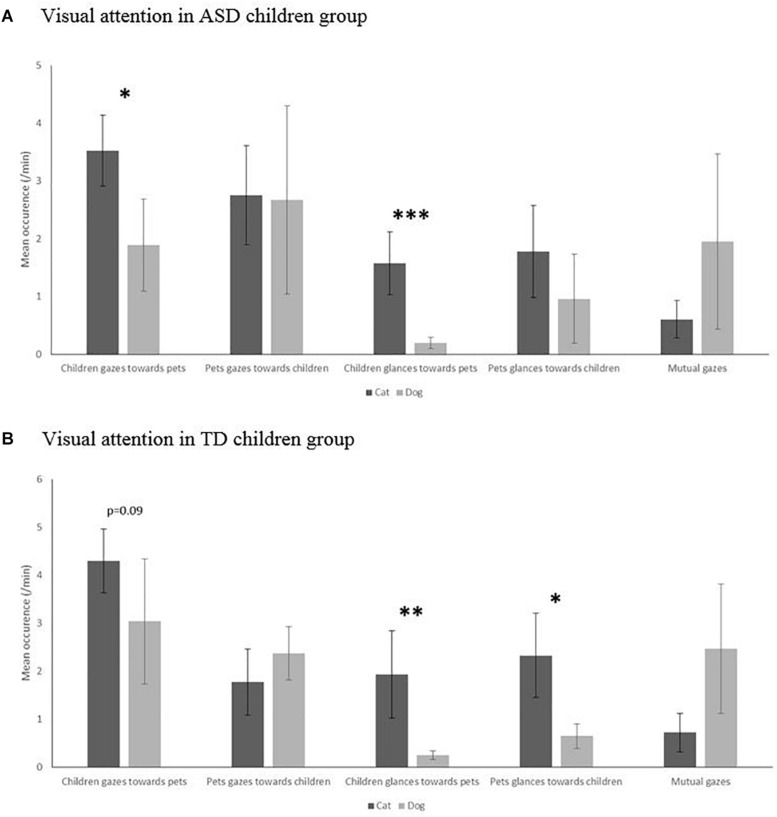
Visual attention in occurrence per minute (glance, gaze, and mutual gaze) in cat–child dyads compared to dog–child dyads in the autism spectrum disorders (ASD) children group **(A)** and typically developing (TD) children group **(B)**. Mann–Whitney level of significance: ^∗^*p* < 0.05, ^∗∗^*p* < 0.01, ^∗∗∗^*p* < 0.001.

The structure of attention clearly differed according to the species in the TD group, with more glances from cats and more gazes from dogs (*U* = 17, *p* = 0.019 and *U* = 11, *p* = 0.007, respectively). On the contrary, no such difference in visual attention according to species could be evidenced in the ASD group (*U* = 29, *p* = 0.103; Figure 2A).

These findings were reflected in the children’s patterns of attention as the TD children also glanced more at cats and gazed more at dogs (*U* = 9, *p* = 0.030 and *U* = 20, *p* = 0.052, respectively) (Figure 2B), whereas ASD children performed more visual attention overall, i.e., more gazes (*U* = 26, *p* = 0.040) and glances (*U* = 4, *p* = 0.0006) toward their cats than their dogs. Interestingly, these observational patterns were not reflected by parents’ reports, which indicated that seven (out of 14, i.e., 50%) children with ASD and nine (out of 12, i.e., 75%) with TD had frequent visual interactions with their pet dogs and that four (30%) children with ASD and five (40%) with TD were considered as often having visual interaction with their pet cats. Thus, both children’s groups were reported as having less visual interactions with cats than with dogs (gazes: *U* = 31, *p* = 0.010; glances: *U* = 10, *p* < 0.001). However, overall, more children were reported as having a privileged relationship with their cat (14/16) than with their dog (17/28) (Fisher’s test: *p* = 0.050). This was especially true for ASD children (8/14 for dogs and 8/8 for cats; Fisher’s test: *p* = 0.040), but less clear for TD children (9/14 for dogs and 6/8 for cats; Fisher’s test: *p* > 0.05).

## Discussion

This study, where dog–child and cat–child spontaneous interactions were observed, shows clear differences in the visual attentional patterns according to the animal species involved: dogs showed more gazes toward children, whereas cats produced both gazes and glances equally. Mutual gazes were rare between children and cats. Children overall produced more gazes than glances toward the animals, whatever the species, and there was no correlation between the attention patterns of children and their pets. Only slight differences were observed according to the developmental status of children: TD children showed longer and more frequent gazes toward their cat than did ASD children. TD child–dog dyads were characterized by more gazes and TD child–cat dyads by more glances. ASD child–dog and ASD child–cat dyads did not differ in terms of attention structure. Overall, ASD children displayed much more visual attention with their pet cats than with their pet dogs and the same amount of visual attention toward their pets, whatever the species, as TD children. Interestingly, parents in both groups reported that their child had few visual interactions with cats as compared to parent reports of children with a dog. However, their reports indicated more bonding with cats than with dogs, especially in the ASD group.

These results confirm that species’ intrinsic characteristics, probably as a result of long-term evolutionary processes, influence the pattern of visual attention in human–animal interactions. Dogs use more visual displays in intraspecific communication, attend to the group members’ intentions through visual cues for social activities, which also allows coordination (Bradshaw and Nott, 1995). Visual communication is an important part of dogs’ social lives, and the repertoire of visual signals is quite diversified, although it has been argued that, as a macrosmatic species, dogs would ignore visual information in some contexts (Szetei et al., 2003; Brucks et al., 2017). Here, our results, where dogs showed more prolonged gazes toward the child than did cats, would rather suggest that, in a pseudo-social context, visual attention is very important, as shown also in the context of intraspecific communication (e.g., Bradshaw and Nott, 1995; Call et al., 2003; Schwab and Huber, 2006; Nagasawa et al., 2015). It might be interesting to compare breeds with differential selections for olfactory skills, but our sample, here, based on opportunistic recruitment, would not allow such comparisons.

Cats, as solitary opportunistic gregarious animals, seem to not only have developed a less varied repertoire of visual signals but also rely less upon visual signals for communicating (Bradshaw and Cameron-Beaumont, 2000). Human children, on the other hand, as many primate species, showed an important pattern of visual attention through gazes toward their pets, although ASD children produced a comparatively more diversified profile. Interestingly, there was no real adjustment within the human–pet dyads and TD children kept showing long gazes to cats, although cats produced more glances than did dogs.

The important visual attention to their pets displayed by ASD children confirms the idea that pets are perceived as potential pseudo-social partners, being less intrusive and “judgmental” than humans. This also confirms that animal faces are less “aversive” than do human faces for these children (Grandin and Johnson, 2005; Grandgeorge et al., 2016). Interestingly, children and their cats were less often seen together than children with pet dogs (a third of the time of observation), which confirms the findings of Hart et al. (2018). Time spent together is also an indication of the type of interactions between the child and the pet (Hart et al., 2018). Despite that, parents indicate that more bonding occurred between the children and cats than dogs. This was especially true for the ASD group, confirming suggestions from Grandgeorge et al. (2012) and Hart et al. (2018) that cats are often more compatible companions.

Although some characteristics of cats, such as accepting being held, displaying “affectionate patterns,” may, in some part, explain these results, there are large individual variations in such behaviors (Mertens, 1991; Hart et al., 2018), which means there may be other features of cats’ behaviors that may explain this attractiveness, especially where ASD children are concerned. One possibility is that the visual attention pattern of cats, with more short glances and less sustained gazes than dogs, may also be more “comfortable” for these children. Recent studies have suggested (1) that direct gaze induces increased arousal in ASD children, this increase being correlated to the degree of social impairment (O’Haire et al., 2015; Kaartinen et al., 2016), and (2) that a less sustained visual attention toward ASD children allows them to be less inhibited and more of the “actor” in the relationship (Grandgeorge et al., 2017). The attention structure of cats, based on repeated glances, may be perceived as less “invasive” and, thus, more favorable for bonding than the long gazes of dogs, especially for ASD children.

Neither dogs nor cats showed a clear difference in their attentional behavior according to the child’s developmental status, although their respective attention structures were more visible with TD children than with ASD children, suggesting some adjustments or modulations by human responses. Overall, the three species involved behaved in the interspecific interactions with their own species-specific visual attention patterning, dogs and TD humans performing more gazes overall during the interactions.

One limitation to this study was of course the length of the videos, which was determined by the ecological situation, but led to limited times of recordings, especially for the cats. Nevertheless, this was a representation of the child–pet relationships. However, this cannot be the sole explanation for the absence of a difference according to species for aspects like mutual gazes as the data, even on these limited samples, were very similar. Further researches should involve longer sampling and should also consider multimodal and complementary aspects of the interactions (e.g., tactile contact and vocal communication).

At that stage, these results strongly suggest, nevertheless, that interspecific interactions, even in the context of human–pet relationships, are highly dependent upon the evolutionary history of the species involved. Because humans rely a lot upon visual communication in their own social encounters, where direct gazes play a major role from early on, they may be especially sensitive to the gazing behavior of their dogs (Nagasawa et al., 2015). People with ASD, with a less typical pattern of interaction, may be more comfortable with the less “invasive” short glances of cats. Pet dogs and cats obviously “project” their own species-specific social skills in the human–animal situation. This means not only that interspecific communication has to be associated with processing and storing other species’ ways of communicating in order to be successful (e.g., Hausberger et al., 2019) but also that the sensitivity to human cues, here the attentional state, demonstrated by different domestic and captive species, is, for a large part, the result of the species’ own evolutionary history.

## Data Availability Statement

The data analyzed in this study is subject to the following licenses/restrictions: Restriction was asked by the participants. Requests to access these datasets should be directed to MG, marine.grandgeorge@univ-rennes1.fr.

## Ethics Statement

Ethical review and approval was not required for the study on human participants in accordance with the local legislation and institutional requirements. Written informed consent to participate in this study was provided by the participants’ legal guardian/next of kin. Ethical review and approval was not required for the animal study because data were collected between 2009 and 2012, in accordance with the (at that time) current French legislation. All dogs and cats involved in the study were family pets, hence under their owners’ responsibility for care and use. The researchers had no involvement in any decision in this regard and the study was conducted in accordance with the French regulations governing the use of animals for research. Written informed consent was obtained from the owners for the participation of their animals in this study.

## Author Contributions

MG and MH designed the experiment, contributed to the statistical analysis, and wrote the manuscript. MG organized the population recruitment. MG, HM, YG, and YB collected the data. MG, HM, YG, YB, and MH performed the analyses. All authors contributed to the article and approved the submitted version.

## Conflict of Interest

The authors declare that the research was conducted in the absence of any commercial or financial relationships that could be construed as a potential conflict of interest.

## References

[B1] Altmann J. (1974). Observational study of behaviour: sampling methods. *Behaviour* 49 227–267. 4597405 10.1163/156853974x00534

[B2] APA (2000). *Diagnostic and Statistical Manual of Mental Disorders 4th edition - revised.* Washington DC: American Psychiatric Press.

[B3] APA (2013). *Diagnostic and Statistical Manual of Mental Disorders*, 5th Edn Washington DC: American Psychiatric Publishing.

[B4] Bekoff M. Daniels T. J. Gittleman J. L. (1984). Life history patterns and the comparative social ecology of carnivores. *Ann. Rev. Ecol. Syst.* 15 191–232. 10.1146/annurev.es.15.110184.001203

[B5] Blois-Heulin C. Girona B. (1999). Patterns of social visual attention in the red-capped mangabey (Cercocebus torquatus torquatus) in the context of food competition. *Folia Primatol.* 70 180–184. 10.1159/000021695 10394071

[B6] Bradshaw J. Cameron-Beaumont C. (2000). “The signalling repertoire of the domestic cat and its undomesticated relatives,” in *The Domestic Cat: The Biology Of Its Behaviour*, eds Turner D. C. Bateson P. (Cambridge: Cambridge University Press).

[B7] Bradshaw J. W. S. Nott H. M. R. (1995). “Social and communication behaviour of companion dogs,” in *The Domestic Dog: Its Evolution, Behaviour and Interactions With People*, ed. Serpell J. (Cambridge: Cambridge University Press), 115–130.

[B8] Brucks D. Soliani M. Range F. Marshall-Pescini S. (2017). Reward type and behavioural patterns predict dogs’ success in a delay of gratification paradigm. *Sci. Rep.* 7:42459. 10.1038/srep42459 PMC5341119 28272409

[B9] Call J. Brauer J. Kaminski J. Tomasello M. (2003). Domestic dogs (Canis familiaris) are sensitive to the attentional state of humans. *J. Comp. Psychol.* 117 257–263. 10.1037/0735-7036.117.3.257 14498801

[B10] Chance M. R. A. Joly C. J. (1970). *Social Groups of Monkeys.* Apes and Men: Jonathan Cape Ltd.

[B11] Day R. L. Coe R. L. Kendal J. R. Laland K. N. (2003). Neophilia, innovation and social learning: a study of intergeneric differences in callitrichid monkeys. *Anim. Behav.* 65 559–571. 10.1006/anbe.2003.2074

[B12] Emery N. J. (2000). The eyes have it: the neuroethology, function and evolution of social gaze. *Neurosci. Biobehav. Rev.* 24 581–604. 10.1016/s0149-7634(00)00025-7 10940436

[B13] Farroni T. Csibra G. Simion F. Johnson M. H. (2002). Eye contact detection in humans from birth. *PNAS* 99 9602–9605. 10.1073/pnas.152159999 12082186 PMC123187

[B14] Forgeot d’Arc B. Delorme R. Zalla T. Lefebvre A. Amsellem F. Moukawane S. (2017). Gaze direction detection in autism spectrum disorder. *Autism* 21 100–107. 10.1177/1362361316630880 27132008

[B15] Goffman E. (1961). *Encounters: Two Studies in the Sociology of Interaction.* London: Mc Millan.

[B16] Goldstein G. Johnson C. R. Minshew N. J. (2001). Attentional processes in autism. *J. Autism. Dev. Disord.* 31 433–440. 11569589 10.1023/a:1010620820786

[B17] Grandgeorge M. Bourreau Y. Alavi Z. Lemonnier E. Tordjman S. Deleau M. (2014). Interest towards human, animal and object in children with autism spectrum disorders: an ethological approach at home. *Eur. Child Adolesc. Psychiatry* 24 83–93. 10.1007/s00787-014-0528-9 24590630

[B18] Grandgeorge M. Degrez C. Alavi Z. Lemonnier E. (2016). Face processing of animal and human static stimuli by children with autism spectrum disorder: a pilot study. *Hum. Anim. Interact. Bull.* 4 39–53.

[B19] Grandgeorge M. Gautier Y. Brugaillères P. Tiercelin I. Jacq C. Lebret M. C. (2017). Social rivalry triggers visual attention in children with autism spectrum disorders. *Sci. Rep.* 7 1–8. 28855550 10.1038/s41598-017-09745-6 PMC5577136

[B20] Grandgeorge M. Tordjman S. Lazartigues A. Lemonnier E. Deleau M. Hausberger M. (2012). Does pet arrival trigger prosocial behaviors in individuals with autism? *PLoS One* 7:e41739. 10.1371/journal.pone.0041739 22870246 PMC3411605

[B21] Grandin T. Johnson C. (2005). *Animals in Translation: Using the Mysteries of Autism to Decode Animal Behavior.* Bloomsbury: Scribner.

[B22] Guellai B. Streri A. Yeung H. H. (2014). The development of sensorimotor influences in the audiovisual speech domain: some critical questions. *Front. Psychol.* 5:812. 10.3389/fpsyg.2014.00812 25147528 PMC4123602

[B23] Guillon Q. Hadjikhani N. Baduel S. Rogé B. (2014). Visual social attention in autism spectrum disorder: Insights from eye tracking studies. *Neurosci. Biobehav. Rev.* 42 279–297. 10.1016/j.neubiorev.2014.03.013 24694721

[B24] Hart L. A. Thigpen A. P. Willits N. H. Lyons L. A. Hertz-Picciotto I. Hart B. L. (2018). Affectionate interactions of cats with children having autism spectrum disorder. *Front. Vet. Sci.* 5:39. 10.3389/fvets.2018.00039 29594156 PMC5862067

[B25] Hattori Y. Kuroshima H. Fujita K. (2007). I know you are not looking at me: capuchin monkeys’ (*Cebus apella*) sensitivity to human attentional states. *Anim. Cogn.* 10 141–148. 10.1007/s10071-006-0049-0 16944232

[B26] Hausberger M. Stomp M. Sankey C. Brajon S. Lunel C. Henry S. (2019). Mutual interactions between cognition and welfare: the horse as an animal model. *Neurosci. Biobehav. Rev.* 107 540–559. 10.1016/j.neubiorev.2019.08.022 31491471

[B27] Kaartinen M. Puura K. Himanen S.-L. Nevalainen J. Hietanen J. K. (2016). Autonomic arousal response habituation to social stimuli among children with Asd. *J. Autism. Dev. Disord.* 46 3688–3699. 10.1007/s10803-016-2908-0 27638648

[B28] Kendon A. (1967). Some functions of gaze-direction in social interaction. *Acta Psychol.* 26 22–63. 10.1016/0001-6918(67)90005-4 6043092

[B29] Kraus C. Waveren C. Huebner F. (2014). Distractible dogs, constant cats? A test of the distraction hypothesis in two domestic species. *Anim. Behav.* 93 173–181. 10.1016/j.anbehav.2014.04.026

[B30] Lemasson A. Blois-Heulin C. Jubin R. Hausberger M. (2006). Female social relationships in a captive group of Campbell’s monkeys (*Cercopithecus campbelli* campbelli). *Am. J. Primatol.* 68 1161–1170. 10.1002/ajp.20315 17096425

[B31] Lemasson A. Hausberger M. (2004). Patterns of Vocal Sharing and Social Dynamics in a Captive Group of Campbell’s Monkeys (*Cercopithecus campbelli campbelli*). *J. Comp. Psychol.* 118 347–359. 10.1037/0735-7036.118.3.347 15482063

[B32] Leroux M. Hetem R. S. Hausberge M. Lemasson A. (2018). Cheetahs discriminate familiar and unfamiliar human voices. *Sci. Rep.* 8 1–6. 30341369 10.1038/s41598-018-33971-1 PMC6195546

[B33] Lord C. Rutter M. Le Couteur A. (1994). Autism Diagnostic Interview-Revised: a revised version of a diagnostic interview for caregivers of individuals with possible pervasive developmental disorders. *J. Autism. Dev. Disord.* 24 659–685. 10.1007/bf02172145 7814313

[B34] MacGrew W. C. (1972). *An Ethological Study of Children’s Behaviour.* New-York and London: Academic Press.

[B35] Maille A. Engelhart L. Bourjade M. Blois-Heulin C. (2012). To beg, or not to beg? that is the question: mangabeys modify their production of requesting gestures in response to human’s attentional states. *PLoS One* 7:e41197. 10.1371/journal.pone.0041197 22815969 PMC3399851

[B36] Mason W. A. (1978). Ontogeny of social systems. *Recent Adv. Primatol.* 1 5–14.

[B37] Mertens C. (1991). Human-Cat interactions in the home setting. *Anthrozoos* 4 214–231. 10.2752/089279391787057062

[B38] Micheletta J. Waller B. M. (2012). Friendship affects gaze following in a tolerant species of macaque, *Macaca nigra*. *Anim. Behav.* 83 459–467. 10.1016/j.anbehav.2011.11.018

[B39] Millot J. L. Filiatre J. C. Gagnon A. C. Eckerlin A. Montagner H. (1988). Children and their pet dogs - how they communicate. *Behav. Process.* 17 1–15. 10.1016/0376-6357(88)90046-0 24896906

[B40] Nagasawa M. Mitsiu S. En E. Ohtani N. Ohta M. Sakuma Y. (2015). Oxytocin-gaze positive loop and the coevolution of human-dog bonds. *Science* 348 333–336. 10.1126/science.1261022 25883356

[B41] O’Haire M. E. McKenzie S. J. Beck A. M. Slaughter V. (2015). Animals may act as social buffers: skin conductance arousal in children with autism spectrum disorder in a social context. *Dev. Psychobiol.* 57 584–595. 10.1002/dev.21310 25913902 PMC8447904

[B42] Sankey C. Henry S. Andre N. Richard-Yris M. A. Hausberger M. (2011). Do horses have a concept of person?. *PLoS One* 6:e18331. 10.1371/journal.pone.0018331 21479184 PMC3068175

[B43] Scaife M. Bruner J. (1975). The capacity for joint visual attention in the infant. *Nature* 253 265–266. 10.1038/253265a0 1113842

[B44] Scheid C. Range F. Huber L. (2007). When, what and whom to watch ? Quantifying attention in ravens (*Corvus corax*) and jackdaws (*Corvus monedula*). *J. Comp. Psychol.* 121 380–386. 10.1037/0735-7036.121.4.380 18085921

[B45] Schwab C. Huber L. (2006). Obey or Not Obey? Dogs (*Canis familiaris*) behave differently in response to attentional states of their owners. *J. Comp. Psychol.* 120 169–175. 10.1037/0735-7036.120.3.169 16893253

[B46] Siegel S. Castellan N. J. (1988). *Nonparametric Statistics for the Behavioral Sciences*, 2nd Edn New York, NY: McGraw-Hill.

[B47] Snowdon C. Hausberger M. (1997). *Social Influences on Vocal Development.* Cambridge: Cambridge University Press.

[B48] Szetei V. Miklósi Á Topál J. Csányi V. (2003). When dogs seem to lose their nose: an investigation on the use of visual and olfactory cues in communicative context between dog and owner. *Appl. Anim. Behav. Sci.* 83 141–152. 10.1016/s0168-1591(03)00114-x

[B49] World Health Organization (1994). *The Composite International Diagnostic Interview, Version 1.1.* Geneva: Researcher’s manual.

[B50] York A. D. Rowell T. E. (1988). Reconciliation following aggression in patas monkeys, Erythrocebus patas. *Anim. Behav.* 36 502–509. 10.1016/s0003-3472(88)80021-6

